# Circulating Levels of Irisin in Hypopituitary and Normal Subjects

**DOI:** 10.1371/journal.pone.0160364

**Published:** 2016-07-29

**Authors:** Lara Pena-Bello, Sonia Pértega-Diaz, Susana Sangiao-Alvarellos, Elena Outeiriño-Blanco, Raquel Eiras-Leal, Bárbara Varela-Rodriguez, Paula Juiz-Valiña, Miguel Pérez-Fontán, María Cordido, Fernando Cordido

**Affiliations:** 1 Department of Medicine, Faculty of Health Sciences, University of A Coruña, A Coruña, Spain; 2 Instituto de Investigación Biomédica (INIBIC), University Hospital A Coruña, A Coruña, Spain; 3 Clinical Epidemiology and Biostatistics Unit, University Hospital A Coruña, A Coruña, Spain; 4 Department of Endocrinology, University Hospital A Coruña, A Coruña, Spain; University of Cordoba, SPAIN

## Abstract

**Context:**

The recently identified myokine irisin conveys some of the benefits of exercise. Hypopituitarism with adult growth hormone deficiency (HP) is a situation characterized by decreased GH secretion and an altered body composition.

**Objective:**

Our aim was to study the skeletal muscle hormone irisin in HP, and compare the results with a similar group of normal subjects.

**Participants and Methods:**

Seventeen HP patients and fifty-one normal subjects of similar age and sex were studied. The diagnosis of GH deficiency was confirmed by the presence of pituitary disease and a peak GH secretion below 3 μg/L after an insulin tolerance test. The patients were adequately treated for all pituitary hormone deficits, except for GH. Fasting serum irisin was measured with an enzyme immunoassay, and HOMA-IR, QUICKI and HOMA-β were calculated.

**Results:**

Fasting irisin levels (ng/ml) were similar in normal [208.42 (168.44–249.23)] and HP patients [195.13 (178.44–241.44)]. In the control group there were moderate significant positive correlations between irisin and BMI, waist circumference, leptin, fasting insulin, HOMA-IR, HOMA-β, triglycerides, and cholesterol. In the control group there were moderate significant negative correlations between irisin and IGF-I and QUICKI. In the hypopituitary group there were moderate significant positive correlations between irisin and body fat and HOMA-β.

**Conclusions:**

We found similar irisin levels in GH deficiency hypopituitary patients when compared with normal subjects. The correlation between irisin and adiposity related factors suggests that that in the case of this clinical model, irisin is regulated by adiposity and not by GH.

## Introduction

Exercise has beneficial effects on systemic metabolism. Fibronectin type III domain-containing type 5 (FNDC5), a type I transmembrane protein, and its circulating form, irisin, conveys some of these benefits in mice [1]. Irisin acts on white adipose cells in culture and in vivo to stimulate uncoupling protein 1 (UCP1) expression and a broad program of brown-fat-like development. Irisin is induced with exercise in mice and humans, and mildly increased irisin levels in the blood cause an increase in energy expenditure in mice, with no changes in movement or food intake [1].

Lean body mass has been found to be the main determining factor for circulating irisin [2]. As in mice, the FNDC5 gene is expressed in human muscle. Age and muscle mass are the primary predictors for circulating irisin, with young male athletes having several-fold higher irisin levels than obese middle-aged women [3]. Circulating irisin levels increase in response to acute exercise, whereas muscle FNDC5 mRNA and circulating irisin levels decrease after surgically induced weight loss in parallel to a decrease in body mass [3]. In contrast, no differences in circulating irisin levels have been found in individuals with sarcopenia compared to control subjects, and were not correlated with the skeletal muscle mass index [4]. Irisin plasma levels have been found to be significantly correlated with high levels of direct and indirect adiposity markers, such as weight, body mass index (BMI), waist circumference, and fat mass, as measured by bioimpedance [5]. Irisin strongly reflects body fat mass, suggesting that circulating irisin levels are conditioned by adiposity levels [6]. Circulating irisin is positively linked to BMI and leptin in school-age children, supporting the notion that irisin is produced by adipose tissue. [7]

Hypopituitarism with growth hormone deficiency (HP) is a clinical situation characterized by decreased GH secretion. In most studies on patients with GH deficiency there is an altered body composition, with decreased lean body mass, which improves with GH treatment [8] [9]. In adults with GH deficiency, lean body mass increased in GH-treated subjects and fat mass was reduced. Changes in lean body mass and fat mass were dose-related [10]. Fifteen-year GH replacement in GH-deficient adults induced a transient decrease in body fat, and sustained improvements of lean soft tissue [9]. In addition to the differences in GH secretion, and taking into account these theoretical differences in body composition in hypopituitary patients, the study of circulating irisin is of major interest in this group of patients. Moreover, recent data suggest a relationship between the GH/IGF-I axis and irisin [11]. Irisin has the potential to be a clinical biomarker of sarcopenia or adiposity in hypopituitarism.

Our aim was to study the skeletal muscle hormone irisin in HP, and to compare the results with a similar group of control subjects.

## Subjects and Methods

### Subjects

All the studies were conducted in accordance with the Declaration of Helsinki. The study protocol was approved by our centre´s ethics committee (Hospital A Coruña, Xunta de Galicia), and written informed consent was obtained from all patients and controls. We included a total of sixty-eight patients and controls in our study. Seventeen HP patients (ten women) were studied. The diagnosis of adult GHD was confirmed by the presence of pituitary disease and a peak GH secretion below 3 μg/L after an insulin tolerance test, at least 12 months prior to the study. The patients were adequately treated for all pituitary hormone deficits, except for GH. The patients were on stable hormone replacement therapy for pituitary hormone deficits in the form of levothyroxine, hydrocortisone, desmopressin, and sex steroids for at least 3 months before joining the study. The adequacy of hormone replacement was assessed at the beginning of the study. None of the patients received GH therapy within 12 months prior to entering the study. The diagnoses of the patient´s pituitary diseases were nonfunctioning pituitary adenoma (n = 6), craniopharyngioma (n = 7), traumatic brain injury (n = 1), nasopharyngeal carcinoma with previous cranial radiotherapy (n = 1) idiopathic empty sella (n = 1), and hypothalamic sarcoidosis (n = 1). As a control group, we studied fifty-one healthy subjects (thirty women), selected from a pool of volunteers available to our unit in a 3:1 ratio. None of the controls had diabetes mellitus or other medical problems, nor were they taking any drugs. The subjects had been eating a weight-maintenance diet and doing their usual exercise routines for at least two weeks prior to the study.

### Study procedure

Between 08.30 and 09.00 a.m., following an overnight fasting and while seated, a peripheral venous line was obtained. Fifteen minutes later we obtained blood samples for irisin, glucose, cholesterol, triglycerides, c-reactive protein, insulin, GH, leptin, and insulin-like growth factor 1(IGF-1). All blood samples were immediately centrifuged, separated and frozen at -80°C. Mid-waist circumference was measured as the midpoint between the iliac crest and the lowest rib, with the patient in the upright position. Total body fat was calculated through bioelectrical impedance analysis (BIA).

### Assays and other methods

All blood samples were immediately differentially centrifuged, and the serum samples obtained were collected and stored at -80 C. Serum irisin was measured with enzyme immunoassay (EIA) kits (Phoenix Pharmaceuticals, Karlsruhe, Germany). The sensitivity of the assay was 6.6 ng/mL and the intra-assay CV was 4%–6%. Serum GH (μg/L) was measured by a solid-phase, two-site chemiluminescent enzyme immunometric assay (Immulite, EURO/DPC, Los Angeles, CA, USA) with a sensitivity of 0.01 μg/L and with intra-assay coefficients of variation of 5.3%, 6.0% and 6.5% for low, medium and high serum GH levels respectively; and with inter-assay coefficients of variation of 6.5%, 5.5% and 6.6% for low, medium and high serum GH levels respectively. IGF-1 (ng/mL) was determined by a chemiluminescence assay (Nichols Institute, San Clemente, CA, USA) and with intra-assay coefficients of variation of 4.8%, 5.2% and 4.4% for low, medium and high serum IGF-1 levels respectively; and with interassay coefficients of variation of 7.7%, 7.4% and 4.7% for low, medium and high serum IGF-I levels respectively. Insulin (μU/mL) was measured with a solid-phase two-site chemiluminescent immunometric assay (Immulite 2000 Insulin, DPC, Los Angeles, CA, USA) and with intra-assay coefficients of variation of 5.5%, 3.3% and 3.7% for low, medium and high serum insulin levels respectively; and with inter-assay coefficients of variation of 7.3%, 4.1% and 5.3% for low, medium and high serum insulin levels respectively. Leptin (ng/mL) was measured by radioimmunoassay (Mediagnost, Tubigen, Germany) and with intra-assay and inter-assay coefficients of variation of 5.3% and 13.6% respectively. Serum glucose (mg/dL) was measured with an automatic glucose oxidase method (Roche Diagnostics, Mannheim, Germany). Serum c-reactive protein, serum triglycerides, serum total cholesterol, serum HDL-cholesterol and serum LDL-cholesterol were measured using the ADVIA Chemistry XPT System (Global Siemens Headquarters, Munich, Germany).All the samples from a given subject were analyzed in the same assay run.

### Calculations

Insulin sensitivity (IS) was measured with the following methods: HOMA-IR with the formula: fasting insulin (μU/mL) x fasting glucose (mmol/L)/22.5; quantitative IS check index (QUICKI) with the formula: 1/[log insulin(μU/mL) + log glucose (mg/dL). For HOMA-IR, lower values indicate higher IS; for QUICKI higher values indicate higher IS [12, 13].

Insulin secretion was estimated using the basal insulin values by the HOMA-β: [20 x fasting insulin (μU/mL)]/[fasting glucose (mmol/L)– 3.5].

### Statistical analysis

Quantitative variables were expressed as median and interquartile range. Due to the small sample size in one of the groups, the non-parametric Mann-Whitney test was used to compare hypopituitary and control subjects with respect to biochemical data, hormonal records and insulin secretion and action indices.

The association between irisin and age, gender, BMI, body fat, waist circumference, fasting glucose, fasting insulin, HOMA-IR HOMA-β, QUICKI, GH, IGF-I, leptin, cholesterol, triglycerides and c-reactive protein was analyzed using Spearman’s Rho correlation coefficient. The linearity of associations with irisin was also explored by means of penalized cubic regression splines.

In the multivariate analysis, both linear regression and generalized additive (GAM) models were used to investigate the variables associated with irisin. Mathematically, some covariates in GAM models can be replaced by arbitrary smooth functions, so finally they were fitted to allow for the nonlinear effects detected in some of the variables studied. Since departures from linearity were not observed for any of the analyzed variables, only the final multivariate linear regression model is shown in the results section.

Statistical analysis was carried out using the R 2.15.1 software (R Foundation for Statistical Computing, Vienna, Austria) and the Statistical Package for Social Sciences version 19.0 for Windows (IBM, Armonk, NY, USA). All statistical tests were two-sided. Only p-values <0.05 were considered statistically significant.

## Results

The age and adiposity indices of the healthy control group and HP patients are shown in Table 1. There was no significant difference in the parameters studied between the two groups.

**Table 1:** Age, BMI, waist and total body fat (median, interquartile ranges) in hypopituitary patients and control subjects.

	Control subjects (n = 51)	Hypopituitary patients (n = 17)
	Median (interquartile ranges)	Median (interquartile ranges)
**Age (years)**	36.00 (30.00–50.00)	39.00 (35.00–46.00)
**BMI (kg/m**^**2**^**)**	27.70 (23.40–31.80)	25.20 (23.00–30.10)
**Waist (cm)**	92.00 (85.00–104.00)	98.00 (90.00–114.00)
**Body fat (%)**	30.00 (24.80–38.20)	27.70 (23.90–36.70)

### Fasting serum levels

Fasting Irisin, fasting glucose, hormones, lipids and c-reactive protein results are shown in Table 2.

**Table 2:** Biochemical and Hormonal data (median, interquartile ranges) in hypopituitary patients and control subjects.

	Control subjects (n = 51)	Hypopituitary patients (n = 17)
	Median (interquartile ranges)	Median (interquartile ranges)
**Irisin (ng/ml)**	208.42 (168.44–249.23)	195.13 (178.44–241.44)
**Fasting Glucose (mg/dl)**	95.00 (89.00–100.00)	92.00 (81.00–103.00)
**Fasting Insulin (μUI/ml)**	6.60 (2.14–11.50)	8.20 (2.00–15.60)
**GH (μg/l)**	0.20* (0.06–1.69)	0.08 (0.05–0.20)
**Cortisol (μg/dl)**	18.00* (15.30–20.70)	2.20 (1.30–5.10)
**IGF-1(ng/ml)**	139.00* (108.00–175.00)	79.00 (60.00–92.00)
**Leptin (ng/ml)**	23.20 (11.50–38.70)	11.90 (6.00–18.70)
**Triglycerides (mg/dl)**	89.00 (66.00–121.00)	96.00 (64.00–126.00)
**Total Cholesterol (mg/dl)**	187.00* (170.00–226.00)	215.00 (198.00–227.00)
**LDL cholesterol (mg/dl)**	105.60 (93.40–138.00)	134.00 (120.00–149.00)
**HDL cholesterol (mg/dl)**	54.00 (46.00–63.00)	54.00 (46.00–59.00)
**C-Reactive Protein (mg/dl)**	0.26* (0.18–0.48)	0.55 (0.30–1.02)
**QUICKI**	0.36 (0.32–0.43)	0.34 (0.31–0.44)
**HOMA-IR**	1.55 (0.56–2.95)	1.96 (0.48–3.97)
**HOMA- β**	79.64 (37.89–130.20)	120.00 (40.00–360.00)

* p<0.05

Fasting Irisin levels were similar in healthy control and hypopituitary patients. Fasting GH levels were lower in the HP group than in healthy controls; 0.08 (0.05–0.20) vs 0.20 (0.06–1.69) for the HP and control group, respectively, p<0.05. Fasting IGF-I levels were lower in the HP group than in healthy controls; 79.00 (60.00–92.00) vs 139.00 (108.00–175.00) for the HP and control group, respectively, p<0.05. Fasting cortisol levels were lower in the hypopituitary group than in healthy controls; 2.20 (1.30–5.10) vs 18.00 (15.30–20.70) for the HP and control group, respectively, p<0.05. Fasting total cholesterol (p<0.05) and c-reactive protein (p<0.05) were higher in the HP group than in healthy controls.

### Insulin secretion and action indices

QUICKI, HOMA-IR and HOMA- β as estimative of insulin action and β cell function (median and interquartile range) are shown in Table 2. QUICKI and HOMA-IR levels were similar in the healthy control and hypopituitary patients. HOMA-β was similar in healthy control and hypopituitary patients.

### Correlations

In the control group there were moderate significant positive correlations between irisin and BMI, waist circumference, leptin, fasting insulin, HOMA-IR, HOMA-β, triglycerides and cholesterol. In the control group, there were moderate significant negative correlations between irisin and IGF-I and QUICKI. In the hypopituitary group, there were moderate significant positive correlations between irisin and body fat and HOMA-β (Table 3).

**Table 3:** Correlations between irisin and the different adiposity indices and hormonal data in the entire group of healthy controls and hypopituitary patients, and in the two individual groups.

	Irisin (ng/ml)			
	Control/ Hypopituitary		Control	Hypopituitary
	Rho	p	Rho	Rho
**Age**	0.111	0.356	0.154	-0.406
**BMI**	0.404	< 0.001	0.412*	0.113
**Waist**	0.311	0.008	0.350*	0.122
**Body fat (%)**	0.343	0.003	0.236	0.537*
**Glucose**	0.141	0.240	0.126	-0.055
**Insulin**	0.408	< 0.001	0.399*	0.448
**GH (μg/l)**	-0.027	0.825	-0.107	0.074
**Cortisol (μg/dl)**	0.074	0.542	0.074	-0.005
**IGF-1(ng/ml)**	-0.163	0.176	-0.331*	0.083
**Leptin (ng/ml)**	0.339	0.004	0.300*	0.369
**Triglycerides (mg/dl)**	0.459	< 0.001	0.558*	0.255
**Cholesterol (mg/dl)**	0.255	0.032	0.349*	0.158
**LDL cholesterol (mg/dl)**	0.129	0.285	0.138	0.162
**HDL cholesterol (mg/dl)**	-0.162	0.177	-0.183	-0.141
**QUICKI**	-0.415	< 0.001	-0.422*	-0.402
**HOMA-IR**	0.415	< 0.001	0.422*	0.402
**HOMA-β**	0.335	0.004	0.324*	0.535*

* p<0.05

There were significant moderate correlations between irisin and BMI, body fat, waist circumference, fasting insulin, HOMA-IR HOMA-β, QUICKI, leptin, cholesterol and triglycerides, in the entire group of healthy controls and HP patients (Table 3). These higher number data have been included for informative purposes only, as correlations including both cohorts grouped together lead to inaccuracies.

**Fig 1:**
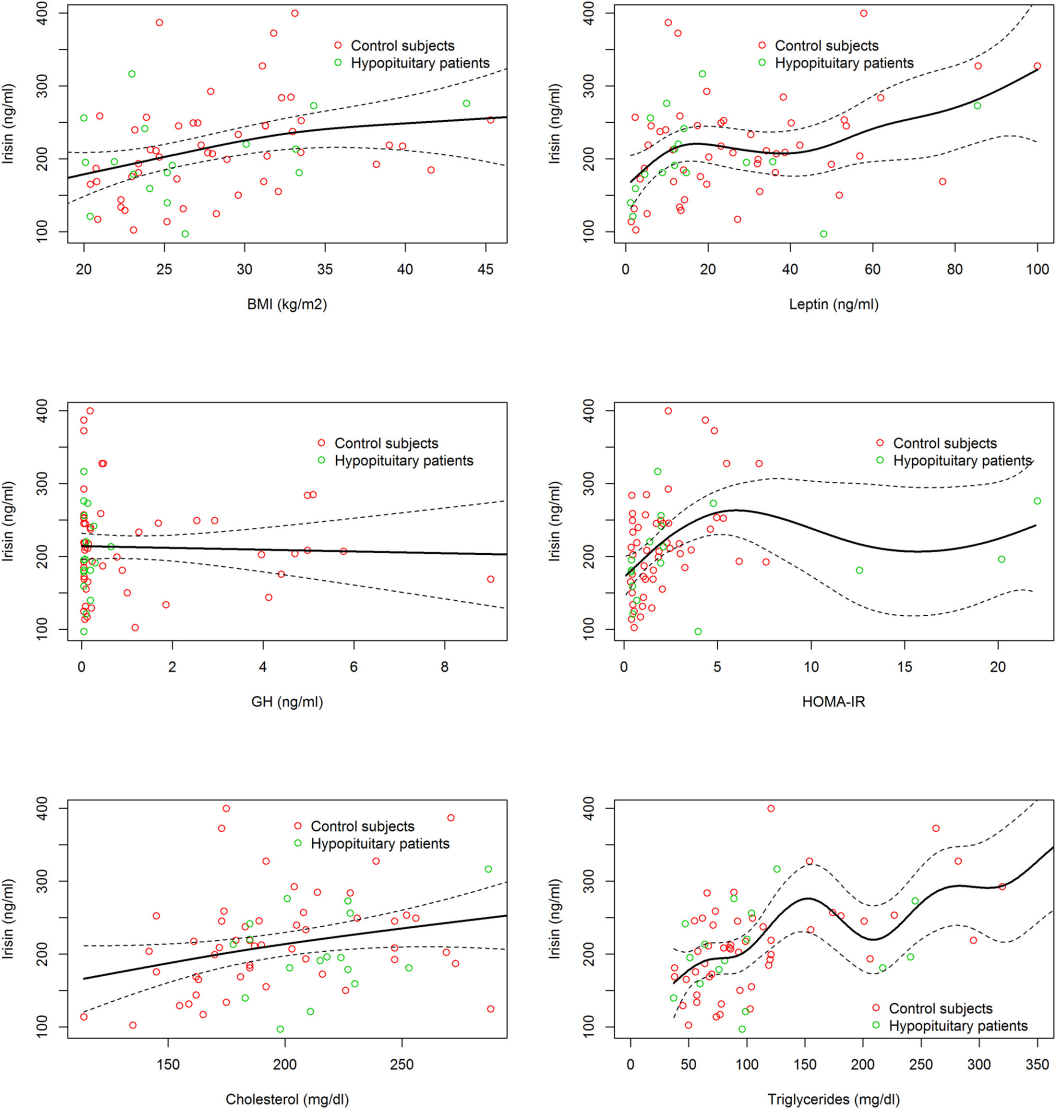
Relationship between Irisin and other parameters by penalized regression splines. Univariate generalized additive regression (GAM) models.

The linearity of associations with irisin was further explored by means of penalized regression splines (Fig 1).

Due to the significant correlations between the adiposity indices, QUICKI, HOMA-IR, HOMA- β and irisin, multivariate analysis was used with both linear regression and multivariate linear regression models in order to quantitatively assess the individual contribution of each predictive measure in explaining the variability between irisin values while in the presence of the remaining predictors.

After adjusting for BMI, HOMA-IR, fasting GH, leptin, cholesterol and triglycerides, only triglycerides continued to be a significant predictor for irisin (Table 4).

**Table 4:** Multivariate analysis of variables related to Irisin values: linear regression model.

	Irisin (ng/ml))				
	B	SE	p	95%CI	
**BMI**	1.725	1.415	0.228	-1.105	4.556
**HOMA-IR**	0.037	2.241	0.987	-4.446	4.519
**Cholesterol**	0.341	0.196	0.088	-0.052	0.734
**Triglycerides**	0.273	0.093	**0.005**	0.087	0.458
**Leptin**	0.527	0.348	0.135	-0.167	1.223
**GH**	1.643	4.167	0.695	-6.692	9.979
**Hypopituitary vs. Control**	-14.581	18.540	0.435	-51.667	22.505

B: Regression coefficient; SE: Standard Error; CI: Confidence interval

## Discussion

The main results of this study were that fasting irisin levels were found to be similar in normal and HP patients. In the control group, there were moderate significant positive correlations between irisin and BMI, waist circumference, leptin, fasting insulin, HOMA-IR, HOMA-β, triglycerides and cholesterol. In the control group, there were moderate significant negative correlations between irisin and IGF-I and QUICKI. In the hypopituitary group, there were moderate significant positive correlations between irisin and body fat and HOMA-β.

Irisin is a 112 aminoacid cleavage product, formed when FNDC 5 is degraded by proteases [1]. It was initially characterized as a myokine, thought to be synthesized and released essentially by the skeletal muscle and the myocardium; however, recent studies have indicated that fat [14] [15] and, to a variable extent, several other peripheral tissues [16] may also secrete this factor. Irisin maintains a remarkable molecular homology across different mammalian species [17].

The mechanisms that regulate irisin synthesis and secretion are not fully understood. Initial suggestions that physical exercise and training could stimulate irisin synthesis and secretion have been supported by the majority of, but not all, later research [16]. Both heat and cold have also been claimed to stimulate irisin release. Serum irisin levels appear to be elevated in obese patients, maintaining a direct correlation with fat mass and body mass index [18], although these associations have not been observed in other studies [16]. It has been posited that irisin levels could be elevated in obese patients as a compensatory mechanism to progressive fat accumulation [18], and also that this hormone could partly mediate the improvement of glucose metabolism observed after weight reduction in obese patients with metabolic syndrome [19]. Relatively low serum irisin levels have been reported in type 2 diabetes [20] [21] and fatty liver disease [18].

This study has revealed a moderate significant positive correlation between Irisin and fasting insulin and HOMA-β. Moreover, there was a moderate significant correlation between Irisin and insulin action indices, such as QUICKI and HOMA -IR. Similar results have been found in other studies, although not in all populations. Serum irisin has been found to be higher and related to insulin in acanthosis nigricans-related obesity [22]. In children, in a multiple linear regression analysis, baseline irisin was significantly associated with HOMA-IR [23]. In women with polycystic ovary syndrome, circulating irisin levels correlated with the area under the curve of insulin secretion and HOMA-IR [24]. In non-diabetic adult subjects, irisin levels were positively correlated with fasting and 2 h post-load insulin levels [25]. In patients with anorexia nervosa and varying stages of obesity, Irisin was positively correlated with BMI, fat mass, body cell mass, fat free mass and insulin levels [26]. In this study, after carrying out a multivariate analysis, only triglycerides continued to be a significant predictor for fasting irisin. Baseline irisin levels have been found to be higher in subjects with metabolic syndrome compared to subjects without metabolic syndrome[27]. In that study, irisin was associated positively with HOMA-IR and triglycerides [27]. In sedentary subjects, irisin levels have been found positively associated with metabolic risk factors including triglycerides [28]. In marked contrast, in obese Chinese adults, stepwise multivariable linear regression analysis showed that fasting insulin was negatively associated with the serum irisin level [29]. The results of our study are in line with those of most of the previous studies, and suggest that circulating fasting irisin is highly dependent on direct and indirect insulin secretion and action indices in control subjects.

We found that fasting irisin levels were similar in HP patients and normal subjects. The HP patients were adequately treated for all pituitary hormone deficits, except for GH, and there were no significant correlations between irisin and either fasting GH or IGF-I, in this group. These data suggest that fasting irisin is not regulated by GH in this clinical model. Nevertheless, we cannot exclude the possibility of GH regulation on irisin in response to acute exercise. In accordance with our results, it has been found that in abdominally obese men with reduced GH that neither serum irisin nor the muscle mRNA expression of FNDC5 changed as a result of exogenous GH treatment [11]. In the HP patients, there were moderate significant positive correlations between irisin and body fat and HOMA-β. In a number of studies, adiposity related factors have been found to be an important regulator of irisin. Irisin levels have been found to be affected by age, sex, obesity, and particularly muscle mass, whereas diurnal rhythm and meals do not contribute to the variation in children and adults [30]. Irisin strongly reflects body fat mass, suggesting that circulating irisin levels are conditioned by adiposity level [5]. Circulating irisin levels increase in response to acute exercise, whereas muscle FNDC5 mRNA and circulating irisin levels decrease after surgically-induced weight loss parallel to a decrease in body mass [3]. Circulating irisin is positively linked to BMI and leptin in school-age children, further supporting again the notion that irisin is produced by adipose tissue [7]. The possibility of a direct interaction between leptin and irisin has been studied, although leptin administration does not alter circulating irisin levels in humans [31]. In line with the previous study, in the HP patients there were no significant correlations between irisin and leptin, suggesting that fasting irisin is not regulated by leptin in this group of patients. Circulating irisin is affected under conditions of altered BMI with the highest levels in severely obese patients. The increase of irisin under conditions of obesity may indicate a physiological function to improve glucose tolerance which is often impaired in obese subjects [26]. Moreover, at tissue level, irisin has been found to be expressed and produced by human muscle and adipose tissue in association with obesity and insulin resistance [32]. The results of our study are in accordance with the previous studies, and suggest that circulating fasting irisin is highly dependent on direct and indirect adiposity related markers in both HP and control subjects. Although the determination of irisin is currently of no clinical relevance in terms of managing hypopituitary patients, in the near future it could be clinically applicable, as it has the potential to be a marker of physical exercise, sarcopenia or adiposity in different clinical situations, including hypopituitarism.

The principal contribution of this work is that, to the best of our knowledge, this is the first study to measure circulating irisin in HP patients. We evaluated circulating irisin in the presence of GH deficiency, with both hormones revealing significant metabolic actions. The present study has several limitations. The number of patients is relatively small, so its statistical power is limited. The presence of a certain degree of partial hypogonadism could be another factor influencing body composition [33]. Moreover, a certain degree of thyroid or adrenal dysfunction cannot be excluded. Nevertheless, the patients were adequately treated for all pituitary hormone deficits, except for GH, and were on stable hormone replacement therapy for pituitary hormone deficits in the form of levothyroxine, hydrocortisone, desmopressin, and sex steroids for at least 3 months before joining the study. The study was not controlled in terms of the amount of exercise carried out, although all of the controls and patients were physically active. None of them were active competitors in sports, and the level of exercise was comparable between controls and HP patients. Moreover, recent data suggest that irisin response to exercise is mainly transient to acute rather than chronic exercise [30, 34, 35]. Doubts have been raised about circulating irisin, and there are limitations due to the irisin assay, although recent data have shown unequivocally that human irisin exists, circulates, and is regulated by exercise [36].

### Conclusions

We found similar irisin levels in GH deficiency hypopituitary patients when compared to normal subjects. The correlation between irisin and adiposity-related factors suggests that in this clinical model, irisin is regulated by adiposity and not by GH.

## Supporting Information

S1 Table Individual numerical data for Irisin, age, BMI, body fat, waist circumference, fasting glucose, fasting insulin, HOMA-IR HOMA-β, quantitative insulin sensitivity check index (QUICKI), GH, IGF-I, leptin, triglycerides, cholesterol, LDL cholesterol, HDL cholesterol and c-reactive protein in hypopituitary and control subjects. (DOCX)
