# Supplementary material for: Circulating Levels of Irisin in Hypopituitary and Normal Subjects
Source: PLoS One. 2016 Jul 29;11(7):e0160364. doi: 10.1371/journal.pone.0160364 (PMC4966893; doi:10.1371/journal.pone.0160364)
Supplement: S1 Table — (DOCX) [file pone.0160364.s001.docx]

| **Nº** | **Age** | **Diagnosis** | **BMI kg/m^2^** | **Waist cm** | **Body fat %** | **Cholesterol mg/dl** | **LDL-Chol mg/dl** | **HDL-Chol mg/dl** | **Triglycerides mg/dl** | **IGF-1 ng/ml** | **Leptin ng/ml** | **CRP mg/dl** | **Glucose mg/dl** | **GH ug/l** | **Insulin uUI/ml** | **Cortisol ug/dl** | **HOMA-BETA** | **HOMA-IR** | **Quicki** | **Irisin ng/ml** |
| --- | --- | --- | --- | --- | --- | --- | --- | --- | --- | --- | --- | --- | --- | --- | --- | --- | --- | --- | --- | --- |
| 1 | 36 | Hypopituitary | 20 | 94 | 14,5 | 228 | 168 | 39 | 104 | 97 | 6 | 1,22 | 106 | 0,06 | 7,6 | 5,1 | 63,63 | 1,99 | 0,34 | 256,06 |
| 2 | 46 | Hypopituitary | 20,1 | 85 | 30,8 | 224 | 134 | 80 | 51 | 54 | 29,4 | 0,38 | 81 | 0,05 | 2 | 2,5 | 40,00 | 0,40 | 0,45 | 195,13 |
| 3 | 42 | Hypopituitary | 23,1 | 86 | 25 | 227 | 158 | 54 | 76 | 148 | 4,7 | 0,41 | 79 | 0,05 | 2 | 17,7 | 45,00 | 0,39 | 0,45 | 178,44 |
| 4 | 49 | Hypopituitary | 33,2 | 105 | 24,8 | 178 | 120 | 50 | 64 | 229 | 11,9 | 0,37 | 104 | 0,65 | 8,2 | 4,2 | 72,00 | 2,11 | 0,34 | 213,13 |
| 5 | 39 | Hypopituitary | 43,8 | 114 | 46,6 | 201 | 131 | 52 | 89 | 30 | 10 | 2,74 | 128 | 0,05 | 69,9 | 1,2 | 387,14 | 22,09 | 0,25 | 275,76 |
| 6 | 42 | Hypopituitary | 26,3 | 120 | 26,3 | 198 | 123 | 56 | 96 | 92 | 48,1 | 3,11 | 103 | 0,05 | 15,6 | 1,2 | 140,40 | 3,97 | 0,31 | 96,56 |
| 7 | 46 | Hypopituitary | 23,8 | 90 | 27,7 | 185 | 90 | 86 | 47 | 83 | 14,2 | 4,68 | 90 | 0,27 | 9 | 2,2 | 120,00 | 2,00 | 0,34 | 241,44 |
| 8 | 35 | Hypopituitary | 21,9 | 85 | 31,6 | 218 | 128 | 42 | 241 | 80 | 35,7 | 0,2 | 103 | 0,08 | 79,4 | 14,9 | 714,60 | 20,19 | 0,26 | 195,82 |
| 9 | 26 | Hypopituitary | 34,3 | 120 | 28,8 | 227 | 144 | 34 | 245 | 210 | 85,5 | 0,59 | 82 | 0,14 | 23,6 | 0,2 | 447,16 | 4,78 | 0,30 | 272,86 |
| 10 | 42 | Hypopituitary | 33,4 | 105 | 36,7 | 202 | 100 | 29 | 475 | 69 | 14,7 | 0,25 | 98 | 0,05 | 52 | 1,2 | 534,86 | 12,58 | 0,27 | 180,98 |
| 11 | 32 | Hypopituitary | 25,5 | 115 | 38,2 | 215 | 149 | 50 | 81 | 82 | 11,9 | 1,02 | 88 | 0,3 | 9 | 12 | 129,60 | 1,96 | 0,34 | 190,82 |
| 12 | 39 | Hypopituitary | 30,1 | 120 | 40,9 | 185 | 108 | 57 | 100 | 42 | 12,8 | 0,58 | 80 | 0,1 | 7 | 10,2 | 148,24 | 1,38 | 0,36 | 219,75 |
| 13 | 35 | Hypopituitary | 23 | 98 | 42,3 | 287 | 203 | 59 | 126 | 75 | 18,7 | 0,55 | 73 | 0,05 | 10 | 2,1 | 360,00 | 1,80 | 0,35 | 316,46 |
| 14 | 34 | Hypopituitary | 25,2 | 100 | 22,7 | 253 | 156 | 54 | 217 | 79 | 8,9 | 0,3 | 81 | 0,2 | 2 | 2,2 | 40,00 | 0,40 | 0,45 | 180,98 |
| 15 | 49 | Hypopituitary | 20,4 | 90 | 13,7 | 211 | 145 | 46 | 99 | 68 | 1,8 | 0,27 | 97 | 0,1 | 2 | 1,3 | 21,18 | 0,48 | 0,44 | 120,88 |
| 16 | 37 | Hypopituitary | 24,1 | 95 | 23,9 | 230 | 149 | 69 | 60 | 50 | 2,4 | 0,25 | 92 | 0,05 | 2 | 5 | 24,83 | 0,45 | 0,44 | 158,82 |
| 17 | 47 | Hypopituitary | 25,2 | 98 | 22,6 | 183 | 71 | 105 | 37 | 60 | 1,2 | 0,61 | 93 | 0,2 | 3 | 1,5 | 36,00 | 0,69 | 0,41 | 139,54 |
|  |  |  |  |  |  |  |  |  |  |  |  |  |  |  |  |  |  |  |  |  |
| **Nº** | **Age** | **Diagnosis** | **BMI kg/m^2^** | **Waist cm** | **Body fat %** | **Cholesterol mg/dl** | **LDL-Chol mg/dl** | **HDL-Chol mg/dl** | **Triglycerides mg/dl** | **IGF-1 ng/ml** | **Leptin ng/ml** | **CRP mg/dl** | **Glucose mg/dl** | **GH ug/l** | **Insulin uUI/ml** | **Cortisol ug/dl** | **HOMA-BETA** | **HOMA-IR** | **Quicki** | **Irisin ng/ml** |
| 18 | 56 | Controls | 28,9 | 106 | 27,4 | 170 | 94 | 52 | 121 | 180 | 32,2 | 0,21 | 105 | 0,78 | 7,1 | 18,7 | 60,86 | 1,84 | 0,35 | 199,07 |
| 19 | 64 | Controls | 31,1 | 92 | 40,8 | 239 | 154 | 54 | 154 | 149 | 100 | 0,6 | 175 | 0,48 | 16,7 | 17,7 | 53,68 | 7,22 | 0,29 | 327,44 |
| 20 | 22 | Controls | 29,6 | 98 | 41,6 | 226 | 148 | 59 | 94 | 122 | 51,9 | 0,58 | 89 | 1,01 | 2 | 21,4 | 27,69 | 0,44 | 0,44 | 149,92 |
| 21 | 56 | Controls | 32,1 | 89 | 38,2 | 192 | 128 | 43 | 104 | 128 | 32,6 | 0,29 | 108 | 0,1 | 7,7 | 20,8 | 61,60 | 2,05 | 0,34 | 155,13 |
| 22 | 42 | Controls | 39 | 120 | 30 | 179 | 105 | 50 | 121 | 101 | 42,3 | 0,25 | 83 | 0,07 | 11,2 | 17,3 | 201,60 | 2,30 | 0,34 | 218,46 |
| 23 | 24 | Controls | 31,1 | 92 | 40,8 | 192 | 90 | 46 | 282 | 130 | 85,6 | 1,37 | 95 | 0,45 | 23,4 | 27,1 | 263,25 | 5,49 | 0,30 | 327,44 |
| 24 | 35 | Controls | 38,2 | 120 | 34,6 | 247 | 200 | 100 | 120 | 148 | 50 | 0,77 | 113 | 0,05 | 27,2 | 9,9 | 195,84 | 7,59 | 0,29 | 192,39 |
| 25 | 41 | Controls | 31,3 | 93 | 36,3 | 247 | 169 | 60 | 92 | 117 | 53,6 | 0,18 | 96 | 0,08 | 8,8 | 14,2 | 96,00 | 2,09 | 0,34 | 245,16 |
| 26 | 35 | Controls | 32,3 | 99 | 42,6 | 228 | 138 | 77 | 66 | 191 | 62 | 0,37 | 85 | 4,98 | 2 | 22,6 | 32,73 | 0,42 | 0,45 | 283,66 |
| 27 | 49 | Controls | 27,9 | 108 | 27,1 | 204 | 90 | 50 | 320 | 72 | 19,7 | 0,53 | 100 | 0,05 | 9,6 | 19 | 93,41 | 2,37 | 0,34 | 292,13 |
| 28 | 57 | Controls | 29,6 | 110 | 43,6 | 209 | 128 | 50 | 155 | 124 | 30,4 | 0,29 | 98 | 1,26 | 2 | 15,1 | 20,57 | 0,48 | 0,44 | 233,06 |
| 29 | 41 | Controls | 31,2 | 95 | 44,8 | 162 | 101 | 47 | 68 | 121 | 77 | 0,13 | 95 | 0,06 | 6,6 | 20 | 74,25 | 1,55 | 0,36 | 168,44 |
| 30 | 32 | Controls | 31,39 | 91 | 43,1 | 142 | 93,4 | 37 | 58 | 108 | 56,9 | 0,77 | 85 | 4,7 | 14,3 | 17,8 | 234,00 | 3,00 | 0,32 | 203,81 |
| 31 | 24 | Controls | 33,13 | 113 | 43,3 | 175 | 96,8 | 54 | 121 | 245 | 57,9 | 0,17 | 93 | 0,19 | 10,3 | 17,7 | 123,60 | 2,37 | 0,34 | 399,47 |
| 32 | 54 | Controls | 28 | 94 | 34,6 | 203 | 124 | 62 |  | 74 | 36,6 | 0,15 | 88 | 5,77 | 8,6 | 15 | 123,84 | 1,87 | 0,35 | 206,95 |
| 33 | 39 | Controls | 31,3 | 87 | 39,4 | 189 | 125 | 53 | 55 | 69 | 17,5 | 1,27 | 94 | 1,69 | 10,3 | 17,6 | 119,61 | 2,39 | 0,33 | 245,44 |
| 34 | 50 | Controls | 34,9 | 109 | 43 | 195 | 114 | 67 | 71 | 85 | 59,2 | 0,24 | 111 | 0,24 | 5,3 | 10,2 | 39,75 | 1,45 | 0,36 | 326,67 |
| 35 | 33 | Controls | 45,3 | 127 | 39,8 | 252 | 171 | 36 | 227 | 54 | 53,1 | 1,05 | 99 | 0,05 | 20,3 | 19,3 | 203,00 | 4,96 | 0,30 | 253,37 |
| 36 | 35 | Controls | 41,6 | 119 | 35,4 | 185 | 125 | 36 | 119 | 81 | 14,1 | 0,4 | 95 | 0,05 | 13,9 | 13,8 | 156,38 | 3,26 | 0,32 | 184,42 |
| 37 | 19 | Controls | 33,5 | 104 | 33,6 | 172 | 104 | 51 | 85 | 123 | 38,7 | 0,29 | 92 | 0,07 | 15,8 | 12,7 | 196,14 | 3,59 | 0,32 | 208,42 |
| 38 | 20 | Controls | 39,82 | 125 | 37,1 | 161 | 102 | 32 | 99 | 185 | 23,2 | 0,2 | 104 | 0,15 | 11,5 | 16,4 | 100,98 | 2,95 | 0,32 | 217,18 |
| 39 | 33 | Controls | 33,51 | 102 | 27,1 | 145 | 87 | 46 | 181 | 118 | 23,8 | 0,48 | 101 | 0,05 | 21,3 | 15 | 201,79 | 5,31 | 0,30 | 252,18 |
| 40 | 28 | Controls | 32,98 | 104 | 22,3 | 183 | 103,2 | 57 | 114 | 228 | 8,4 | 0,1 | 100 | 0,2 | 18,7 | 7,5 | 181,95 | 4,62 | 0,31 | 237,21 |
| 41 | 34 | Controls | 31,8 | 105 | 25,8 | 173 | 69 | 51 | 263 | 85 | 12,7 | 0,28 | 96 | 0,05 | 20,4 | 20,7 | 222,55 | 4,84 | 0,30 | 372,24 |
| 42 | 50 | Controls | 21 | 74 | 27,6 | 174 | 113 | 46 | 73 | 89 | 13,2 | 0,28 | 93 | 0,42 | 2 | 15,3 | 24,00 | 0,46 | 0,44 | 258,48 |
| 43 | 37 | Controls | 20,8 | 80 | 30 | 181 | 118 | 55 | 38 | 212 | 11,7 | 0,12 | 89 | 9,01 | 5,2 | 20,4 | 72,00 | 1,14 | 0,38 | 168,44 |
| 44 | 56 | Controls | 24,7 | 75 | 29,1 | 269 | 178 | 72 | 93 | 94 | 20,2 | 0,26 | 95 | 3,96 | 2 | 23,4 | 22,50 | 0,47 | 0,44 | 202,14 |
| 45 | 33 | Controls | 23 | 84 | 32,6 | 145 | 87 | 47 | 56 | 184 | 18,2 | 0,1 | 89 | 4,4 | 2 | 16,7 | 27,69 | 0,44 | 0,44 | 175,32 |
| 46 | 50 | Controls | 27,1 | 85 | 24,7 | 256 | 171 | 73 | 62 | 101 | 40,2 | 0,19 | 97 | 2,54 | 8,2 | 18,5 | 86,82 | 1,96 | 0,34 | 249,23 |
| 47 | 40 | Controls | 27,3 | 91 | 19,8 | 185 | 93 | 33 | 295 | 175 | 5,5 | 0,48 | 96 | 0,05 | 2,9 | 19,8 | 31,64 | 0,69 | 0,41 | 218,46 |
| 48 | 36 | Controls | 25,9 | 90 | 16,5 | 173 | 95 | 38 | 201 | 141 | 6,2 | 0,2 | 96 | 0,05 | 7,3 | 18 | 79,64 | 1,73 | 0,35 | 245,16 |
| 49 | 42 | Controls | 23,9 | 85 | 18,5 | 208 | 135 | 38 | 174 | 133 | 2,3 | 0,15 | 104 | 0,05 | 4,5 | 20,6 | 39,51 | 1,16 | 0,37 | 256,97 |
| 50 | 43 | Controls | 26,8 | 95 | 32,5 | 231 | 156 | 54 | 105 | 146 | 23,2 | 0,54 | 94 | 2,93 | 2 | 15,2 | 23,23 | 0,46 | 0,44 | 248,93 |
| 51 | 34 | Controls | 22,3 | 80 | 24,8 | 162 | 88 | 63 | 57 | 159 | 14,3 | 0,25 | 81 | 4,12 | 5,2 | 11,3 | 104,00 | 1,04 | 0,38 | 143,54 |
| 52 | 36 | Controls | 23,4 | 88 | 26,7 | 185 | 101 | 76 | 38 | 176 | 36,4 | 0,22 | 97 | 0,9 | 6,6 | 18,5 | 69,88 | 1,58 | 0,36 | 180,76 |
| 53 | 53 | Controls | 23,4 | 102 | 30,4 | 209 | 122 | 46 | 206 | 170 | 32 | 0,74 | 110 | 0,23 | 22,7 | 11,2 | 173,87 | 6,17 | 0,29 | 193,30 |
| 54 | 27 | Controls | 24,7 | 110 | 23,8 | 271 | 148 | 43 | 398 | 130 | 10,4 | 0,27 | 98 | 0,05 | 18 | 27 | 185,14 | 4,36 | 0,31 | 386,98 |
| 55 | 32 | Controls | 22,3 | 77 | 25,3 | 175 | 105,6 | 58 | 57 | 201 | 13,1 | 0,1 | 91 | 1,86 | 2,14 | 16,4 | 27,51 | 0,48 | 0,44 | 133,76 |
| 56 | 35 | Controls | 22,56 | 85 | 34,5 | 155 | 76 | 70 | 45 | 157 | 13,4 | 0,17 | 96 | 0,22 | 6,23 | 18,95 | 67,96 | 1,48 | 0,36 | 128,81 |
| 57 | 38 | Controls | 24,14 | 75 | 28,9 | 190 | 64 | 96 | 86 | 130 | 11,5 | 0,3 | 79 | 0,09 | 2 | 20,2 | 45,00 | 0,39 | 0,45 | 212,38 |
| 58 | 29 | Controls | 20,42 | 75 | 28,6 | 163 | 78 | 68 | 48 | 178 | 19,7 | 0,2 | 71 | 0,14 | 2 | 24,3 | 90,00 | 0,35 | 0,46 | 164,72 |
| 59 | 19 | Controls | 20,86 | 76 | 28,3 | 165 | 94,6 | 55 | 77 | 238 | 27,2 | 0,18 | 63 | 0,12 | 5,62 | 21,6 | 130,20 | 0,88 | 0,39 | 116,83 |
| 60 | 51 | Controls | 23,18 | 78 | 29,3 | 205 | 107,8 | 83 | 71 | 133 | 9,7 | 0,18 | 92 | 0,2 | 3,36 | 14,5 | 41,71 | 0,76 | 0,40 | 239,74 |
| 61 | 57 | Controls | 27,7 | 85 | 36,1 | 247 | 156 | 75 | 80 | 172 | 26,1 | 0,18 | 92 | 4,98 | 5,4 | 17,7 | 67,03 | 1,23 | 0,37 | 207,93 |
| 62 | 57 | Controls | 24,5 | 87 | 38,7 | 187 | 121 | 53 | 67 | 139 | 34,2 | 0,42 | 98 | 0,14 | 10,2 | 24,6 | 104,91 | 2,47 | 0,33 | 210,88 |
| 63 | 52 | Controls | 20,76 | 89 | 17,5 | 273 | 163 | 61 | 64 | 93,1 | 4,6 | 0,17 | 102 | 0,47 | 4,25 | 22 | 39,23 | 1,07 | 0,38 | 186,60 |
| 64 | 27 | Controls | 23,1 | 94 | 21,5 | 135 | 82 | 43 | 50 | 164 | 2,5 | 0,36 | 108 | 1,18 | 2,06 | 19,2 | 16,48 | 0,55 | 0,43 | 101,93 |
| 65 | 37 | Controls | 28,25 | 93 | 19 | 288 | 210,4 | 57 | 103 | 139 | 5,2 | 0,18 | 110 | 0,05 | 2 | 15,3 | 15,32 | 0,54 | 0,43 | 124,35 |
| 66 | 28 | Controls | 25,18 | 77 | 12,7 | 114 | 65 | 55 | 74 | 158 | 1,4 | 0,16 | 82 | 0,07 | 2 | 17,4 | 37,89 | 0,40 | 0,45 | 113,71 |
| 67 | 30 | Controls | 26,19 | 90 | 20,9 | 159 | 97 | 56 | 78 | 139 | 2,1 | 0,24 | 105 | 0,09 | 3,8 | 17,6 | 32,57 | 0,99 | 0,38 | 131,26 |
| 68 | 28 | Controls | 25,79 | 91 | 20,5 | 216 | 134 | 68 | 70 | 203 | 3,5 | 0,74 | 85 | 0,06 | 4,95 | 23,4 | 81,00 | 1,04 | 0,38 | 172,25 |

**Table S1.** Individual numerical data for Irisin, age, BMI, body fat, waist circumference, fasting glucose, fasting insulin, HOMA-IR HOMA-β, quantitative insulin sensitivity check index (QUICKI), GH, IGF-I, leptin, triglycerides, cholesterol, LDL cholesterol, HDL cholesterol and c-reactive protein in hypopituitary and control subjects
